# The recognition of dental anxiety. A comment on ‘Dental clinicians recognizing signs of dental anxiety: a grounded theory study’ by M. Höglund, I. Wårdh, S. Shahnavaz and C. Berterö

**DOI:** 10.1080/00016357.2023.2277252

**Published:** 2024-03-26

**Authors:** Helen R. Chapman, Nima Moghaddam, Nick Kirby-Turner

**Affiliations:** School of Psychology, University of Lincoln, Brayford Pool, Lincoln, LN6 7TS, UK; Clinical Psychologist, West Sussex, UK

We read the article, ‘Dental clinicians recognizing signs of dental anxiety: a grounded theory study’ by Höglund and colleagues [1] with interest and would like to offer some observations.

We welcome the research approach taken as it has been helpful in eliciting and systemising the hypotheses. This has enabled the development of a helpful construct – the clinical eye – which calibrates the process by which dental clinicians describe and categorize some elements of their identification of dental anxiety in real-life clinical practice. The authors comment that the clinical eye is ‘shorthand’ and omits some important aspects and defining features of a more complete understanding of the nature of dental anxiety.

The clinicians in the study used signs of sympathetic activation as an objective proof of dental anxiety. The authors rightly highlight that individuals may not display external signs of anxiety for a variety of reasons [2–4]. However, another significant reason is that the three major aspects of fear (behavior or avoidance, reported or cognitive fear, and physiological arousal) may be desynchronous or variably dominant in the individual’s presentation as fearful and, importantly, they may change at different rates [5,6]. It is our experience that cognitive fears are usually the last to change; one can be treating a completely co-operative, apparently relaxed patient, who suddenly uses the stop signal and then careful inquiry is necessary to elucidate the fear.

Therefore, we suggest that the ‘clinical ear’ is also crucial for identifying and responding appropriately to dental anxiety: i.e. clinicians must harness active listening skills to elicit and fully engage with expressions of cognitive fear - attending compassionately to verbal (in addition to non-verbal) cues from patients. These fears may be viewed by clinicians as ‘unrealistic’ (p. 5) but are very real to the patient. It is important that patients who report significant fears are believed, and it is unfortunate that some clinicians chose to disbelieve the patients unless they were showing signs of physiological arousal or cooperation difficulties (p. 7); this is classic desynchrony and the authors are correct when they state that this may lead to distrust of the dentist.
